# Tyrosinase Immobilization Strategies for the Development of Electrochemical Biosensors—A Review

**DOI:** 10.3390/nano13040760

**Published:** 2023-02-17

**Authors:** Alexandra Virginia Bounegru, Constantin Apetrei

**Affiliations:** Department of Chemistry, Physics and the Environment, Faculty of Sciences and the Environment, “Dunărea de Jos” University of Galați, 47 Domnească Street, 800008 Galați, Romania

**Keywords:** enzymatic activity, biosensor, cross-linking, entrapment, selectivity

## Abstract

The development of enzyme biosensors has successfully overcome various challenges such as enzyme instability, loss of enzyme activity or long response time. In the electroanalytical field, tyrosinase is used to develop biosensors that exploit its ability to catalyze the oxidation of numerous types of phenolic compounds with antioxidant and neurotransmitter roles. This review critically examines the main tyrosinase immobilization techniques for the development of sensitive electrochemical biosensors. Immobilization strategies are mainly classified according to the degree of reversibility/irreversibility of enzyme binding to the support material. Each tyrosinase immobilization method has advantages and limitations, and its selection depends mainly on the type of support electrode, electrode-modifying nanomaterials, cross-linking agent or surfactants used. Tyrosinase immobilization by cross-linking is characterized by very frequent use with outstanding performance of the developed biosensors. Additionally, research in recent years has focused on new immobilization strategies involving cross-linking, such as cross-linked enzyme aggregates (CLEAs) and magnetic cross-linked enzyme aggregates (mCLEAs). Therefore, it can be considered that cross-linking immobilization is the most feasible and economical approach, also providing the possibility of selecting the reagents used and the order of the immobilization steps, which favor the enhancement of biosensor performance characteristics.

## 1. Introduction

The selective determination of small amounts of analytes in complex systems can be difficult, and therefore, there is still a pressing need to develop simple and accessible analytical techniques [1] and to select the most suitable nanomaterials for building sensitive devices [2,3].

Biosensors can be considered as alternatives to traditional analytical methods (e.g., high performance liquid chromatography or spectrophotometry) due to their unique characteristics, e.g., fast response, miniaturization, relatively low cost and the possibility of real-time monitoring of some parameters. These advantages make biosensors suitable for analyses in the food industry, the environment or medicine [4].

A biosensor is a device composed of an immobilized biologically sensitive element (e.g., enzyme, DNA, antibody) that recognizes an analyte (e.g., chemical compound, complementary DNA, antigen) and a transducer, which is used to convert the (bio)chemical signal resulting from the interaction of the analyte with the bioreceptor into an electrical signal. Furthermore, biosensors using bioassays and microbial biosensors are also used to detect various compounds of interest [2,5].

**Figure 1:**
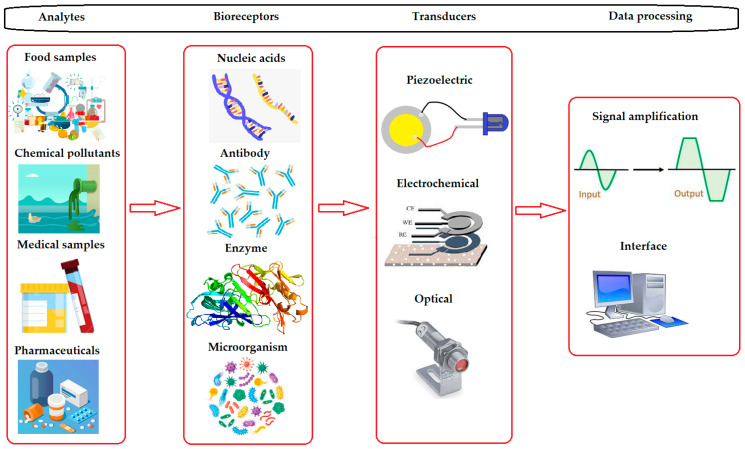
Components of a typical biosensor.

Enzymes are most commonly used as a biosensitive element that is immobilized on electrodes of different types [4,6]. The intensity of the generated signal is directly or inversely proportional to the analyte concentration. Figure 1 shows a schematic of the components of a biosensor.

In the development process of a biosensor, the main performance criterion is maintaining bioreceptors (e.g., enzymes) on the electrode surface and the accessibility of their active centers, avoiding their degradation in the biosensor construction process [6,7,8,9,10].

A multitude of methods and protocols by which the enzyme is immobilized by the transducer have been presented in the literature over the years [11]. These include physical adsorption [12], covalent binding [13], affinity binding [14], immobilization using three-dimensional matrices (silica gel or carbon paste) [15,16], by cross-linking [17,18] or by several combined immobilization strategies [19,20]. The choice of immobilization technique is made taking into account the advantages and disadvantages of each approach (Table 1) but also the nature of the immobilized enzyme, the transducer used and the electrochemical method of detection [21].

Another criterion for the selection of the immobilization strategy is the scope of the biosensor, namely, whether the priority is sensitivity or maximum stability. The reproducibility, cost and difficulty of the immobilization process should also be carefully evaluated [22].

The performance of biosensors has also been effectively improved by incorporating carbonaceous nanomaterials on the transducer surface [23,24], as well as through the use of metallic and bimetallic nanoparticles [25], redox mediators [26] or ionic liquids [27], i.e., elements that increase conductivity and electron transfer between the active centers of the enzyme and the electrode surface. Moreover, direct immobilization of the enzyme, e.g., by electrodeposition, without the use of prior adsorption on metal nanoparticles, can lead to the denaturation of the sensitive element and a loss of bioactivity [28].

The type of enzyme to be immobilized on the electrode surface is chosen based on the type of sample and the chemical structure of the analyte. Enzymes catalyze different reactions that are fundamental to a wide range of biological functions, such as gene regulation, cell communication, energy conversion (photosynthesis and respiration), drug purification and cell defense [29]. 

Among the enzymes studied for applications in enzyme-based biosensors, tyrosinase is one of the most important, because it has good versatility as a biocatalyst of the oxidation process of different phenolic compounds of interest in food, medicine, pharmacy, etc. Therefore, this work aims to perform a critical analysis of the main tyrosinase immobilization strategies using (or not) electron transfer mediators for the development of the most efficient tyrosinase-based electrochemical biosensors.

## 2. Tyrosinase—Sources and General Characteristics

According to the international classification proposed by the International Union of Biochemistry and Molecular Biology, enzymes are classified into six classes based on the type of catalyzed reaction: oxidoreductases, transferases, hydrolases, lyases, ligases and isomerases. An enzyme code (EC) is assigned to each enzyme according to this classification. In the oxidoreductases class (EC 1), there are polyphenol oxidases (PPO), i.e., enzymes that contain copper and catalyze the oxidation of phenolic compounds to o-quinones and favor secondary polymer reactions for the formation of melanins and reticulated fibers. PPOs are divided into (1) tyrosinases, (2) catechol oxidases and (3) laccases. These classifications are based on substrate specificity and their mechanism of action. Tyrosinases (EC 1.14.18.1) have cresolase and catecholase activities; catechol oxidases catalyze the oxidation of o-diphenols to o-quinones, and laccases catalyze the oxidation of phenolic compounds, carrying electrons that are used in the reduction of molecular oxygen to water [30,31].

**Figure 2:**
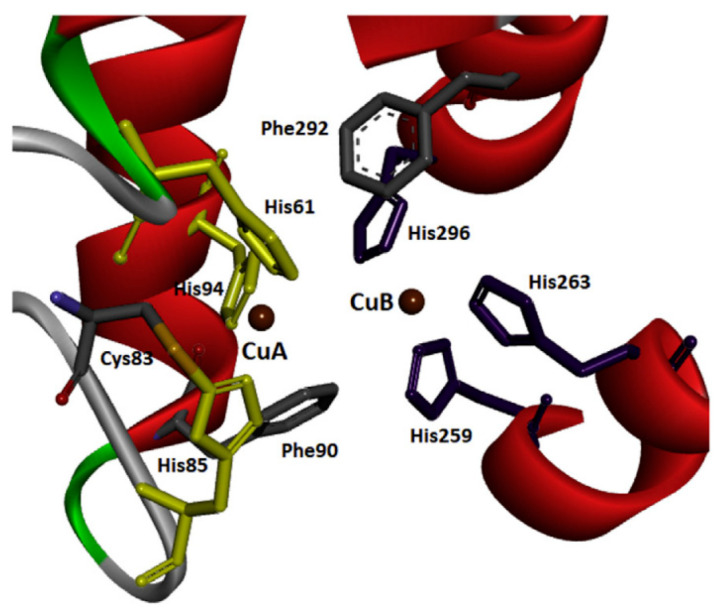
Geometry of the binuclear copper-binding site for isolated and purified tyrosinase from *Agaricus bisporus*. Reprinted with permission from [35]. Copyright 2023, copyright Elsevier.

Tyrosinases have been isolated and purified from different sources, i.e., some plants, animals and microorganisms. A major and cheap source of tyrosinase that closely resembles human tyrosinase is the fungal species *Agaricus bisporus* [32]. Tyrosinase from *Agaricus bisporus* is a 120 kDa tetramer with two different subunits, one heavy and one light [33]. It was first isolated in 1895 by Bourquelot and Bertrand [34]. Figure 2 shows the geometry of the binuclear copper-binding site for tyrosinase isolated and purified from *Agaricus bisporus*. 

Structurally, tyrosinases (also called monooxygenases) have three domains and two copper-binding sites that bind to six histidine residues and interact with molecular oxygen at the active site of tyrosinase. They also have a disulfide bond that stabilizes their structure [36]. 

**Figure 3:**
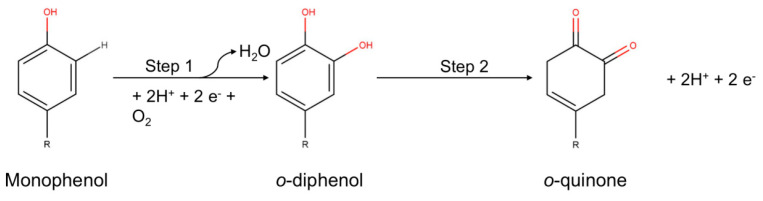
Reactions catalyzed by tyrosinase.

The binuclear copper center is capable of introducing oxygen at the ortho position of an aromatic ring to an existing hydroxyl group [37,38]. These enzymes catalyze the conversion of monophenols (e.g., tyrosine) to o-diphenols, followed by the oxidation of the o-diphenols to the corresponding o-quinone derivatives (diphenolase activity) (Figure 3) [39,40,41]. 

The active site of tyrosinase contains copper, which is found in the catalytic cycle in three active forms in the stationary phase, depending on its oxidation state: met-tyrosinase in the form Cu^+2^Cu^+2^, deoxy-tyrosinase in Cu^+1^Cu^+1^ form and oxy-tyrosinase with peroxide in the active site Cu, Cu^+2^Cu^+2^ and the inactivated form deact-tyrosinase with Cu°Cu^+2^ [39].

In the case of dopamine, the end product of the tyrosinase-catalyzed reaction is dopaquinone, which is a precursor of melanin [40,41]. All three oxidation states of tyrosinase (oxy-, met- and deoxy-tyrosinase) are involved in melanin formation, depending on the binuclear structures of copper in the active site [42].

Melanin is the main pigment primarily responsible for pigmenting people’s skin, hair and eyes. It is produced by melanocytes through melanogenesis [43]. Although melanogenesis is a complicated process represented by numerous enzymatic and chemical reactions, enzymes such as tyrosinase and other tyrosinase-related proteins (TYRP1 and TYRP2) play an important role in melanin synthesis, catalyzing several steps of this process [44]. Tyrosinase is also the main cause of the excessive browning of fruits and vegetables, as well as diseases resulting from melanin overproduction. 

Therefore, control of enzyme activity by tyrosinase inhibitors is an essential effort for the treatment of some dermatological conditions but also for possible benefits in the food industry [36]. The cosmetic industry is developing products that reduce skin pigmentation spots by acting on tyrosinase [45]. In-depth studies on enzyme activity can prevent unwanted browning of fruit to obtain better quality end products. For example, in the case of virgin olive oil, color and flavor are indicators of quality, which is influenced by the concentrations of phenolic compounds in the composition [46,47,48,49,50,51]. 

For the identification and study of effective tyrosinase inhibitors, it is necessary to know the different sources of the enzyme. The source of tyrosinase influences its structure and functions [50].

As mentioned above, in fungal tyrosinases, a histidine residue is linked by a thioether linkage to the side chain of a cysteine residue. This feature is not found in bacterial tyrosinase [50]. Some researchers have reported that there are some similarities but also differences between tyrosinase binding sites of different origins using the same target molecules [51]. Comparing the characteristics of tyrosinases from fungi and plants, a number of differences emerge. There are studies suggesting that the enzymes exhibit different characteristics in terms of substrate specificity, stereospecificity, inhibition and ability to bind to the model protein, while nonetheless showing similar reaction mechanisms for the formation of identical quinone radicals [52]. 

Although monophenols and diphenols bind and orient identically at the active site, only the monophenols rotate during the reaction. This allows differentiation between enzymes with both mono- and diphenolase activity and those with only diphenolase activity. Basically, for there to be only diphenolase activity, two constraints must coexist in the enzyme (e.g., the one in sweet potato) to prevent mono-phenolase activity. These relate to the chemical structure of the enzyme, specifically, the presence of a bulky residue above the active site (e.g., a bulky phenylalanine residue) and a thioether binding restriction on the active site histidine. The presence of these structural features prevents the rearrangement of the substrate necessary for the hydroxylation of monophenolase but allows the oxidation of a diphenol [53]. 

### Tyrosinase Inhibition

In general, tyrosinase inhibitors are screened in the presence of a monophenolic substrate such as tyrosine or a diphenolic substrate such as L-dopa, and activity is assessed based on the formation of dopachrome [36].

There are two ways in which tyrosinase activity can be truly inactivated, namely [54]:

- Specific tyrosinase inactivators or self-destructive inactivators. This inactivation may be explained by conformational changes, triggered by the substrate and then mediated by solvent molecules, in the tertiary and quaternary structures of tyrosinase [55]. The chemical structure of different substrates is diverse (o-diphenols, ascorbic acid, aminophenols and o-diamine hydroxyhydroquinone), but the process always requires an oxidation-reduction step [56,57,58,59,60,61].
- “true inactivators”, which can have several mechanisms: competitive, non-competitive and mixed. A competitive inhibitor can bind to a free enzyme and thus avoid binding of the substrate to the active site of the enzyme. Such compounds are copper chelators, e.g., aromatic acids (D-tyrosine [60]), phenolic and polyphenolic compounds and some non-aromatic compounds (sulfonamides—such as sulphanilamide, cyanides—such as potassium cyanide, hydroxamic acids—such as benzohydroxamic acid, iodide ions—such as potassium iodide and nitrates—such as nitroglycerin) [50].

On the other hand, a non-competitive inhibitor can only bind to the enzyme-substrate complex (e.g., thiazole derivatives [61]), while a mixed inhibitor (competitive and non-competitive mixed) can bind to both the free enzyme and the enzyme-substrate complex (e.g., phthalic acid and hydroxypyridinone derivatives of cinnamic acid [62,63].

Despite the existence of a wide range of natural (a range of phenolic compounds) and synthetic tyrosinase inhibitors (glabridin [64]), few have been intensively studied and are considered safe. Therefore, in vivo and in vitro studies are needed for these tyrosinase inhibitors to be used in the food, cosmetic or pharmaceutical industries. 

## 3. Tyrosinase-Based Electrochemical Biosensors—Characteristics 

In an electrochemical biosensor, the target molecule is determined by converting the biochemical reaction signals into an electrical signal: voltage, current, impedance, etc. There are several types of electrochemical biosensors classified and described below based on the detection principle [65].

Amperometric biosensors provide accurate quantitative analytical information depending on the amount of current resulting from the redox reaction under a constant potential applied to it. The signal from the recognition element varies before and after interaction with the analyte. The sensitivity of the amperometric biosensor is determined by comparing the intensity of the current obtained for different analyte concentrations. These biosensors have two electrodes: one for applying the voltage and the other for measuring the current passing through the device [66]. 

Voltametric biosensors are another type of electrochemical biosensor that uses voltametric techniques (cyclic voltammetry, differential pulse voltammetry, square wave voltammetry); they obtain information by varying the potential and then measuring the resulting current. Biosensors measure the current-potential relationship in the form of voltametric curves. The potential where the redox peaks appear is specific for the analyte, and the current peak magnitude is proportional to the concentration of it.

Impedance biosensors determine analytes or biologically active molecules with the help of impedance variation. Impedimetric biosensors measure the variations in charge conductance and capacitance at the biosensor surface as the selective binding of the analyte take place. The output of an electrical impedance signal will be proportional to the activity of the target analyte [67]. 

Potentiometric biosensors have a potential difference between the working and the reference electrode as their operating principle. Potentiometric biosensors measure the potential difference between electrodes at zero current value. The measured species are not consumed as they are the case of amperometric biosensors. The response is proportional to the concentration of the analyte, i.e., by comparing its activity with the reference electrode [68].

The ideal biosensor should be sensitive (to determine the analyte concentration according to the applications specification), selective, compact, easy to use and provide an accurate and fast response regardless of sample temperature and pH. Furthermore, the fabrication process must be reproducible and have reasonable cost. 

The development of enzyme biosensors has successfully overcome various challenges such as enzyme stability, loss of enzyme activity, response time and the possibility of using them at different stages of the production process. In general, biosensors have various applications in the medical and pharmaceutical fields, as well as in the food industry and environmental applications [69]. 

Tyrosinase-catalyzed oxidation of phenolic compounds is important in the food industry, e.g., to obtain the typical flavor of tea or coffee [70]. 

In the analytical field, tyrosinase is used to develop biosensors that exploit its ability to catalyze a wide range of phenolic compounds. The detection and quantification of phenols with high sensitivity, selectivity and accuracy are required in many areas [45]. 

With new generation biosensors, a more sensitive response is obtained by reducing the electroactive products to quinone, and the enzymatic reaction can be evidenced by a cathode current. Quinones can be reduced directly [71,72] or by redox mediators with catalytic activity [73,74], usually at a negative potential [45]. The intensity of the cathodic current is proportional to the concentration of phenolic compounds present in the sample. 

It is worth noting that the performance of the electrodes is obtained in the optimal potential range for electrochemical measurements, which is −0.2–0 V. In this potential range, interference is less likely, background noise is lower and the biosensor can detect lower concentrations of analyte [45]. The pH of the electrolytes and solutions to be analyzed is an important parameter for the stability and reproducibility of tyrosinase-based biosensors. Most studies show that the most stable and well evidenced responses were obtained at pH 7.0 [73,74,75]. The literature shows diverse configurations of tyrosinase-based biosensors with very good results, low detection limits and extended linearity ranges [17,33,76,77,78]. 

Tyrosinase can also be immobilized by various methods on several types of support electrodes (glassy carbon electrode, carbon paste electrode, screen-printed electrode, etc.) [76,79,80,81,82,83,84], each having advantages and limitations in preparation. For example, in the case of a carbon paste electrode, the enzyme can be incorporated into the paste (which contains a mineral oil) [85], or it can be immobilized beforehand in the preparation step of the graphite powder [45]. However, the main problem with this type of electrode is its low stability. This limitation could be overcome if quasi-solid binders, such as paraffin wax, were used in the preparation of the paste, which could better protect the electrode material [83,84,85].

Furthermore, different nanomaterials can be added to improve the performance of biosensors (e.g., carbon nanotubes, graphene, carbon quantum dots, metal nanoparticles) [75,76,78,80,86,87,88]. For example, metal nanoparticles (NPs) lend themselves very well to association with tyrosinase because their structure, shape and size can be exploited for optimal immobilization of the enzyme. Moreover, NPs exhibit biocompatibility, low cytotoxicity but a high capacity for direct electron transfer between the metal centers of the enzyme and the electrode [20,88,89,90,91,92,93].

Nanoparticles, nanofibers, nanotubes and nanocomposites exhibit high surface area and mechanical properties that allow for efficient enzyme delivery with minimal diffusion limitation [94], as well as a large volume of enzyme [95]. 

## 4. Tyrosinase Immobilization Strategies 

Through immobilization, enzymes increase their stability and efficiency for practical applications [96]. Immobilization can be defined as the attachment of the enzyme to a support (electrode) using organic (i.e., natural and synthetic polymers) [97] or inorganic (natural minerals and processed materials) materials [98]. 

It should be recognized that tyrosinase may undergo changes in chemical and physical properties following immobilization, depending on the choice of immobilization method. An important influence on the stability of the enzyme is the supporting matrix and its kinetic properties. Additionally, the surface on which the enzyme is immobilized is responsible for maintaining the tertiary structure of the enzyme through the formation of electronic transition complexes or the formation of hydrogen or covalent bonds with the matrix [99].

Therefore, when aiming at the immobilization of tyrosinase on a surface, the main objective is to preserve the catalytically active tertiary structure of the enzyme to maximize its stability and reactivity after immobilization [100,101]. 

Three fundamental factors to be considered when immobilization of tyrosinase is desired are: the selection of the support electrode and modifier nanomaterials, the immobilization conditions and the analyte to be detected.

The characteristics of the support material are very important for the effectiveness of the immobilized enzyme. The properties (mechanical, chemical, biochemical and kinetics) of the support material, together with the properties of the enzyme, determine the properties of the biocomposite system formed [102]. The properties of an ideal carrier material would be hydrophilicity, inertness to enzymes, biocompatibility, antimicrobial resistance, compressive strength and affordability [103].

Even if there is no universal carrier that is suitable for all enzymes and applications, certain characteristics of the carrier material should be taken into account, such as high affinity for proteins, availability of reactive functional groups, mechanical stability, rigidity, feasibility of regeneration, low toxicity and biodegradability [104].

**Figure 4:**
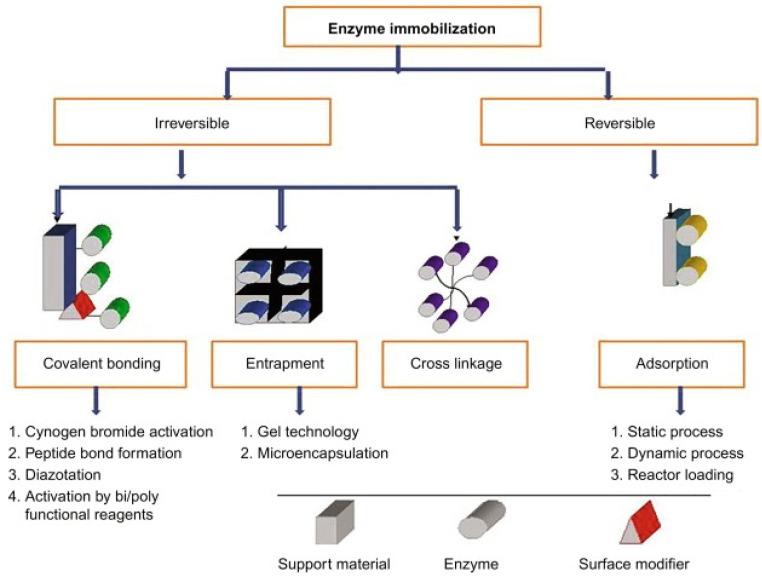
Classification and representation of different enzyme immobilization techniques. Reprinted with permission from [105]. Copyright 2023, copyright Elsevier.

There are various approaches to classifying immobilization techniques, one of which is based on the reversibility/irreversibility of enzyme binding to the support/adsorbent material. Furthermore, immobilization methods can also be classified according to the physical or chemical method used for immobilization [105,106]. The classification is shown in Figure 4.

Physical or reversible methods involve:

(1) adsorption
(2) formation of disulphide bonds.

Chemical or irreversible methods involve:

(1) covalent bonding,
(2) reticulation and
(3) capture.

This section will detail the main techniques for tyrosinase immobilization using various nanomaterials with the aim of maintaining the catalytic activity of the enzyme and achieving stable, accurate and rapid responses. The advantages, disadvantages as well as challenges arising from each strategy will be discussed. In addition, ways to improve biosensor performance by these immobilization methods, and combinations thereof, will be highlighted.

### 4.1. Physical Adsorption

Adsorption is a simple (i.e., does not require complex or expensive equipment or processes), reversible technique in which the enzyme is immobilized by mixing with an adsorbent compound and attaching it to the support material under appropriate conditions and subsequently detaching the loosely bound proteins by washing after an appropriate incubation time. Adsorption between the enzyme molecule and the carrier matrix can take place via hydrogen bonds, van der Waals forces, ionic bonds or covalent bonds [105]. These forces can also act simultaneously, depending on the structure of the enzyme with which they interact. The attractive force is usually weak and does not affect the enzyme topography or activity. Other types of interactions between the enzyme and the associated solid carrier are also involved, mainly caused by electrostatic and hydrophobic forces [107].

These forces are influenced by the enzyme molecule, the surface of the support material, water and other compounds present in the mixture [108].

Van der Walls or London dispersion forces, although weak, imply a non-specific physical interaction between the enzyme and the surface of the support material, influenced by certain external factors, such as large amounts of reactants, temperature variations, pH and high ionic strength [109].

Reversibly immobilized enzymes can be removed from the support material under mild conditions when the enzyme activity has degraded, and the support can be reused for further enzyme adsorption. This is an economic advantage, as the price of the support material represents a significant percentage of the total cost of the device [99,110].

Enzymes can be immobilized by physical adsorption involving compounds acting as “carriers”. A carrier molecule can be used to immobilize an enzyme molecule if there is a corresponding affinity between the two, reflected by the presence of active groups on the carrier surface, the particle size and the pore structure [105]. Such compounds can be inorganic ones, such as silica, gold, titanium, alumina gel, zirconia gel or bentonite. Depending on the enzyme properties, a certain pore size of inorganic nanoparticles is preferred. Studies show that the enzyme immobilized in macropores is more active than that in mesopores [111].

Among the organic compounds used as carriers are chitin, starch, alginate, collagen, polyacrylamide and polyurethane.

These compounds are considered supports for enzyme adsorption by preparing some types of membranes, e.g., porous hollow fiber membranes, cotton flannel membranes and nylon membranes [112].

Immobilization or non-covalent adsorption can be reversed if the pH, ionic strength, temperature or solvent polarities change. The method involves binding the enzyme to the substrate of the carrier molecules, avoiding physical and chemical modification of the enzyme. 

Sometimes, in physical adsorption, a modifying agent is also used to facilitate the immobilization process through the active functional groups in the molecule. Basically, some of the functional groups anchor chemically to the carrier while others interact physically with the enzyme.

To summarize, the literature reports several methods by which the enzyme can be immobilized by adsorption, such as:

(a) a static process involving physical contact between the enzyme molecule and the carrier compound, with no dynamics involved.
(b) a dynamic process involving the adherence of the enzyme to the carrier by mechanical agitation, even for commercial production of enzymes in large reactors.
(c) by electrodeposition as follows: the carrier is placed near the electrodes immersed in the enzyme solution, and by passing the current, the adhesion of the enzyme to the carrier compound is initiated [105].

Since the structure of the enzyme can influence the adsorption process, it is important to describe and analyze the tyrosinase adsorption techniques reported in the literature.

For example, a dynamic physical adsorption process has been reported in a recent study for the immobilization of tyrosinase on the surface of a supported composite material formed by the electrodeposition of polycaprolactone and chitosan. Basically, tyrosinase was immobilized by creating mixed ionic bonds and hydrogen bonds between the enzyme and the prepared composite material. After optimizing the optimal immobilization conditions, the morphology of the fibers and the presence of the enzyme were confirmed by SEM imaging, and the immobilization yield and enzyme activity were above 90%. In this case, tyrosinase, through the developed system, was able to biodegrade bisphenol by more than 80% within 120 min. The collected experimental data demonstrated that the stability and reusability of tyrosinase were significantly improved by this immobilization technique.

**Figure 5:**
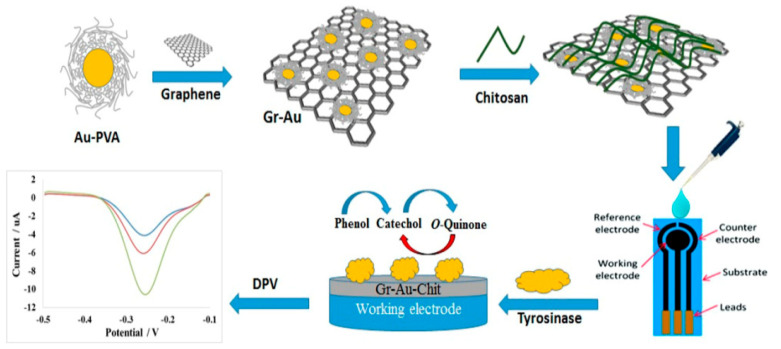
Construction of the electrochemical biosensor [113].

Chitosan and gold nanoparticles were used to immobilize tyrosinase also by physical adsorption in Fuzi Mohamed Fartas et al.’s [113] study to build an electrochemical biosensor for the detection of phenolic compounds. In the first step, a graphene–gold nanocomposite was synthesized. In the preparation of the nanocomposite, polyvinylalcohol acts as a protective ligand to prevent agglomeration of Au nanoparticles, and NaBH_4_ acts as a reducing agent. After synthesis, the nanocomposite was dispersed in deionized water and sonicated. The chitosan solution was prepared separately. The two solutions were mixed in volume ratio of 3:1. After dispersion of the Gr-Au-Chit mixture on the SPCE surface, the previously prepared tyrosinase solution was added. Figure 5 shows the possible mechanism for the synthesis of the Gr-Au nanocomposite and the fabrication of an electrochemical biosensor based on Gr-Au-Chit/Tyr. The formation strategy of Gr-Au involved the creation of hydrophilic interactions via polyvinyl alcohol. The next step ensured electrostatic binding between chitosan having a positive charge of amino -NH_3_^+^ groups and PVA stabilized gold nanoparticles with the negative charge of hydroxyl -OH groups. Subsequently, the enzyme was immobilized on the surface of the modified electrode by physical adsorption. The observed electrochemical signal was attributed to the reduction of o-quinone to catechol formed during enzyme catalysis on the electrode surface.

The characterization of the biosensor by FESEM and EDX confirmed the immobilization of the enzyme on a large surface of the biosensor, having a homogeneous, porous and smooth morphology, indicating that the biosensor was properly prepared. The results revealed good selectivity and reproducibility of the biosensor due to the excellent conductivity and large active surface area generated by the Gr-Au nanocomposite. In addition, the developed biosensor demonstrated the potential applicability for monitoring phenolic compounds in environmental settings [113].

In Appan Roychoudhury’s [114] study, Ni oxide nanoparticles were used as carrier molecules for tyrosinase immobilization by physical adsorption. The newly constructed biosensor aimed to detect dopamine in real samples by electrochemical methods. Nickel oxide (NiO) nanoparticles were synthesized by the sol-gel method using an anionic surfactant, while sodium dodecyl sulfate (SDS) was used to adjust the nanoparticle size. Structural and morphological studies of the prepared NPs were performed using X-ray diffraction (XRD), transmission electron microscopy (TEM) and dynamic light scattering (DLS) techniques. Subsequently, tyrosinase was immobilized by physical adsorption on the surface of NiO NPs. After Fe_3_O_4_ adsorption, the enzyme-coated NPs were deposited on a flexible polyethylene terephthalate (PET) substrate coated with indium tin oxide (ITO) by the drop and dry method. The enzyme-NP nanocomposite was investigated by atomic force microscopy (AFM) and Fourier transform infrared spectroscopy (FTIR). Dopamine determination was performed with high sensitivity and selectivity over a wide linear range of detection. Validation of the biosensor by detection of dopamine in real samples confirmed the effectiveness of the enzyme immobilization strategy [114]. 

Another interesting approach [115] is the construction of mesoporous magnetic microspheres formed from Fe_3_O_4_ and silica (Fe_3_O_4_@mSiO_2_), modified with tyrosinase by cross-linking. This biosensitive device has been used for the electrochemical detection of phenol from various real samples. Following the surface characterization by transmission electron microscopy, a high immobilization capacity of the enzyme and a proper adhesion to the electrode surface could be observed without the use of an additional adhesive. The silica coating on the magnetic core gives the material a more easily functionalized surface and prevents the aggregation of microspheres. Optimization of the mesoporous silica coatings resulted in suitable particle sizes and optimal porosity, demonstrated by N_2_ adsorption/desorption experiments. The porous structure increases surface roughness, which facilitates enzyme immobilization by increasing the amount of and maintaining adequate enzyme activity. The superparamagnetic properties of Fe_3_O_4_@mSiO_2_ microspheres offer the possibility of redispersion in solution and reuse. The stability of the magnetic nanocomposite on the surface of the magnetic electrode depends on the physical adsorption, magnetic interaction, magnetic nanocomposite adhesion and gravitational and wash-off forces that can cause their removal. The selectivity of the biosensor was evaluated by detecting phenol in the presence of an interfering substrate. The results showed that these interferents do not considerably influence the biosensor response [115].

Another tyrosinase immobilization strategy was reported by Wang Yue. It involves physical adsorption of the enzyme onto a carbon pellet, together with the acridine orange dye. Co-adsorption of the dye was essential to prevent degradation of the enzyme on the carbon surface. The developed biosensor was used for sensitive amperometric detection of toxic chlorophenols, but it may also have applications for bioreactors or combustion biopiles [116].

Recently, Yan Zhang et al. improved this method of physical co-adsorption by using a mineral such as molybdenite instead of carbon. Molybdenite, together with the same dye (acridine orange), were of particular importance for optimizing the physical adsorption of tyrosinase on the surface of a glassy carbon electrode. Molybdenite plays an important role as a ligand and stabilizer of the adsorbed enzyme, and the dye increases the catalytic activity of tyrosinase, thus considerably improving the biosensor signal [117].

### 4.2. Covalent Bonding

This method is based on the formation of a covalent bond between a chemical used as a carrier and an enzyme with a nucleophilic group [118]. This technique requires prior activation of the support using a coupling agent with multifunctional groups (e.g., hydroxyl, amino, imino, thiol, imidazole). Subsequently, the enzyme creates covalent bonds to the activated surface of the support, and then excess or remaining unbound biomolecules are removed by washing. Common covalent binding methods include alkylation, diazotization, silanation, Schiff’s base formation, thiol-disulfide, peptide bond formation and amidation [119]. Covalent binding provides benefits such as strong bond formation and high stability; however, conformational modification of the enzyme structure decreases enzyme activity [120]. The strong interaction between the enzyme and the “carrier” that occurs during the catalytic reaction makes immobilization irreversible [121,122].

For example, certain polymers can covalently bond with biomolecules without the need for prior surface modification or cross-linking. These polymers are usually strong electron acceptors, which can react rapidly at physiological pH values. Copolymers containing cyclic structures, such as poly(glycidylmethacrylate-co-methacrylate) (P(GMA-co-MA)) and poly(styrene-co-maleic anhydride) (PSMA), are usually used for the attachment of biomolecules. PGMA has ring groups along the polymer backbone. Epoxies tend to be more electrophilic due to this strained ring system. The amine groups present on the biomolecules attack the electrophilic carbon next to the epoxy oxygen, resulting in a negative charge on the oxygen and a positive charge on the nitrogen. The extra electron pair of oxygen removes a hydrogen from the ammonium nitrogen, yielding an alcohol group and an amide group [123,124].

Unfortunately, this technique only works for a select few polymers that contain an epoxy group, limiting its use. Although researchers have grafted epoxy-containing groups onto polymers, these systems do not result in similar pH tolerance, showing mainly only temperature tolerance [124].

If the desired nanofiber sublayer does not contain a strong electrophilic grouping, such as epoxies, surface modifications can be made to introduce the desired functional groups onto the fiber surface. Polyacrylonitrile (PAN) is a widely used polymer in various industries due to its good physical characteristics and ease of electrospinning [125,126]. For enzyme immobilization, PAN is used for its high mechanical strength and thermal resistance [127,128]. However, due to the inertness and the hydrophobicity of the acrylonitrile monomer, functional groups must be introduced onto the polymer surface through an amidination reaction. Amidation reactions result in imidoester functional groups reacting with primary amines to form amidine bonds.

Covalent binding causes electron sharing between the amine functional group of the enzyme molecule and the surface of the support [129]. This technique prevents the loss of enzyme from the surface of the carrier compound, even under unfavorable reaction conditions [109]. However, the complexity of the method, the risk of decreased enzyme activity and the toxicity of cross-linking reagents (when cross-linking is used) may be limitations that need to be considered.

In the study by Jessica Rocha Camargo et al. [130], the covalent binding of tyrosinase had as its substrate a homogeneous film made of carbon nanodiamonds and potato starch (ND-PS) in an optimized ratio. The biosensor formation aimed at determining catechol using differential pulse voltammetry in river and tap water. After modification of the glassy carbon electrode with ND-PS, a solution containing 1-ethyl-3-[3-dimethylaminopropyl] carbodiimide (EDC), N-hydroxysuccinimide (NHS) and phosphate buffer (pH = 6.6) was dispersed on the active surface of the sensor. 

In this case, 1-ethyl-3-(3-dimethylaminopropyl) carbodiimide (EDC) acts as a cross-linking agent. It is a water-soluble carbodiimide that will react with the carboxyl groups on the ND-PS substrate [131]. The carboxyl groups and EDC interact, forming an active O-acylidene intermediate compound that can be easily displaced by a nucleophilic attack from the primary amino groups of the target material.

**Figure 6:**
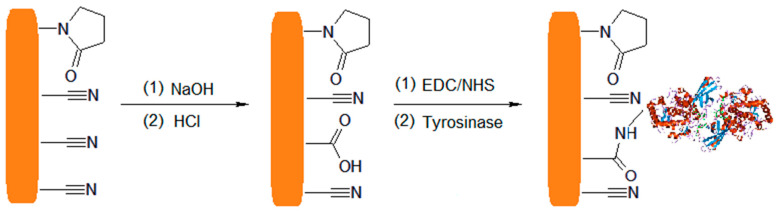
Reactions occurring between sublayer, capture agent and enzyme.

In most studies, EDC is not used alone but together with N-hydroxysuccinimide (NHS). NHS is a capture agent that improves the reaction yield [132]. If EDC alone were used, there is a risk of forming undesired intermediates, such as N-acylureas, which modify the structure of the molecule, inactivating certain portions [133]. NHS rapidly interacts with the unwanted intermediate, forming reactive esters, thus avoiding side reactions. Subsequently, it will react with the amine on the biological molecule by covalent binding of an acyl group to the nucleophile with the release of the leaving group of the NHS [134]. The reaction between the EDC and the NHS is a zero-length cross-linking reaction, since cross-linking agents facilitate the binding between the enzyme and the sublayer polymer but do not remain present in the final compound [135]. From the hydrolysis in a basic environment (NaOH) followed by neutralization with HCl, a partial conversion of nitrile groups (–C≡N) into carboxyl (–COOH) groups occurs, which are then activated by EDC/NHS, becoming useful for the immobilization of tyrosinase. These reactions are schematically represented in Figure 6.

**Figure 7:**
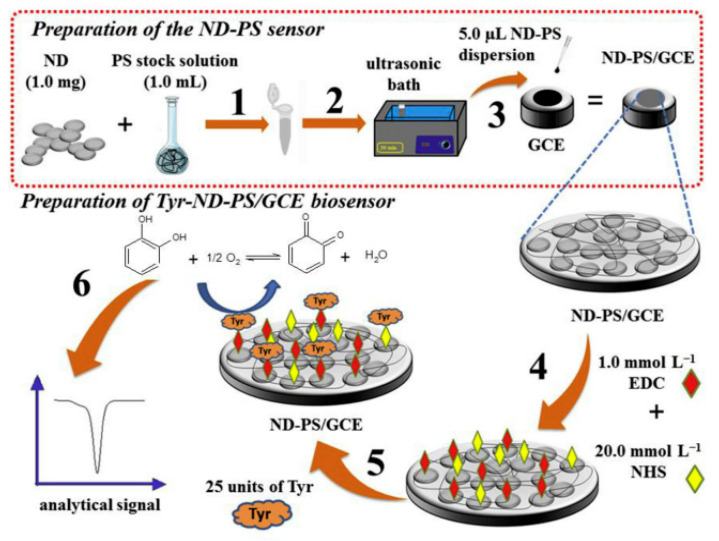
Schematic of ND-PS/GCE and of Tyr-ND-PS/GCE preparation. Step 1: ND was dispersed in PS solution. Step 2: ND-PS dispersion was ultrasonicated (30 min). Step 3: A volume of 5.0 µL ND-PS were dropped on the GCE surface. Step 4: ND-PS/GCE was immersed in a solution containing 1-ethyl-3-[3-dimethylaminopropyl] carbodiimide (EDC) and N-hydroxysuccinimide (NHS) for 2 h. Step 5: ND-PS/GCE was immersed in a solution containing 25 units of Tyr. Step 6: Detection of catechol in samples of tap and river water with the new biosensor. Reprinted with permission from [130]. Copyright 2023, copyright Elsevier.

A schematic of the development of the biosensitive platforms built by Jessica Rocha Camargo can be seen in Figure 7.

After the construction of the biosensor, its morphology was explored by SEM, TEM, EDX, SAED and FTIR, making it possible to observe the homogeneity and uniformity of the biopolymer, the agglomeration of NDs in PS, the covalent immobilization of the enzyme and the lack of contaminants and crystallinity in the ND structure. The efficient detection limit and electroanalytical performance confirmed the practical utility of the biosensor and the success of covalent immobilization of tyrosinase using the polymer [130].

Another study reported a new biosensor in which tyrosinase was covalently attached to the electrode surface modified with gold nanoparticles via N-(3-dimethylaminopropyl)-N’-ethylcarbodiimide hydrochloride and N-hydroxysulfosuccinimide (EDC/NHSS). 

Initially, the screen-printed electrode was modified with gold nanoparticles by electrodeposition and then immersed in 3-mercaptopropionic acid solution for the formation of a carboxylic acid monolayer on the AuNP surface. Subsequently, tyrosinase solution (1 mg/mL in 50 mM phosphate buffer solution pH 6.5) was then added onto the activated EDC/NHSS. This resulted in random covalent binding of the amine fragments of tyrosinase to the carboxyl group on the AuNP-modified electrode surface. Cyclic voltammetry and square-wave voltammetry have been applied for the selective determination of phenol from regional waters in South Korea, with promising results [136]. 

A recent paper [137] described the electropolymerization and characterization of a polymeric film of 4-mercaptophenylacetic acid to create covalent bonds between the carboxyl groups (left unchanged after electropolymerization by the sulfur atom) on the sensor surface and the tyrosinase subsequently attached by immersion of the electrode in the enzyme solution. The enzyme biosensor was used for the detection of tyramine. Similar to previous work, the carboxyl groups were activated by immersing the electrode in a solution consisting of n-hydroxysuccinimide (NHS), 1-ethyl-3-(3-dimethylaminopropyl) carbodiimide hydrochloride (EDC) and 2-(N-morpholino) ethanesulfonic acid (MES), forming covalent bonds with the enzyme via a nucleophilic acid substitution reaction. Without activation of the carboxyl groups in the formed polymer, morphological characterization shows the presence of electrostatic or weak Van der Waals-type interactions. Optimization of the parameters (pH, enzyme concentration) led to the formation of covalent bonds between enzyme and polymer and to a robust enzyme biosensor with high sensitivity and affinity for tyramine and efficient reproducibility in wine samples [137].

Another important cross-linking agent is glutaraldehyde (Glu), a saturated dialdehyde used in a variety of applications and fields. Glu cross-links amine-containing biomolecules, but its mechanism of action is not fully known due to uncertainties in the chemical structure and polymer size [138]. It is considered that in aqueous media, depending on pH, Glu could be a monomer, a dimer or a polymer. For enzyme immobilization by glutaraldehyde, rigorous optimization of parameters such as pH, concentration, reaction time and temperature is required [139].

Similar to EDC/NHS, Glu can be used as a cross-linking agent with or without surface treatment, depending on the functional groups present on the substrate. Like NHS, glutaraldehyde reacts with amine-rich substrates such as natural polymers (proteins) or polyamides [140,141,142].

This is how Zhen Yong Lu proceeded in their study to develop an amperometric biosensor for catechol detection. Covalent binding of tyrosinase to the surface of a poly(thionine) modified glassy carbon electrode by electrodeposition was achieved by glutaraldehyde. Prior to enzyme immobilization, poly(thionine) was deposited on the electrode surface by the electro-oxidative polymerization of thionine, followed by cross-linking of terminal amino groups with the lysine protein group (or cysteine group) by Glu, which was confirmed by SEM evaluation. The resulting TYR/GA/pTN/GCE biosensor showed a fast and sensitive response to catechol and a detection limit of 6.0 µM. The biosensor retained 71% of its initial activity for catechol oxidation after one month of storage [143].

**Figure 8:**
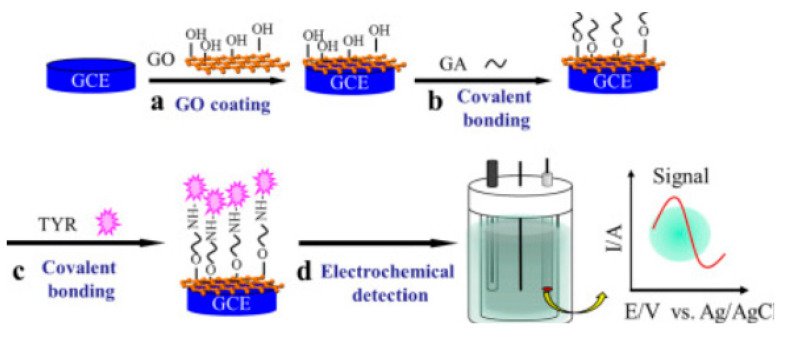
Schematic illustration of the TYR/GA/GO/GCE biosensor for the electrochemical determination of phenol derivatives: (**a**), GO casting; (**b**), covalent bonding of GO and GA; (**c**), covalent bonding of GA and TYR; and (**d**), electrochemical detection of phenol derivatives. Reprinted with permission from [80]. Copyright 2023, copyright Elsevier.

A considerable advantage of Glu is that it can also react with hydroxyl groups to form acetal bonds [139]. Thus, Glu can bind with both the -NH_2_ groups in the polymeric compound and the -OH groups in the graphene oxide, e.g., creating multiple pathways for tyrosinase immobilization. This technique was proposed by Yue Wang in 2018 for the development and characterization of a modified graphene oxide biosensor for the determination of phenolics (Figure 8). First, graphene oxide (GO) was deposited on the surface of a glassy carbon electrode. Then, glutaraldehyde cross-linked the OH groups on the graphene oxide surface. Finally, the enzyme was immobilized on the Glu/GO-modified surface. The steps of the development of the new biosensor are shown in Figure 8. The GO matrix provides the functional groups which are essential for the subsequent covalent binding of the enzyme. FE-SEM confirmed tyrosinase self-assembly with a snowflake-like morphology. Under optimized conditions, the TYR/GA/GO/GCE biosensor exhibited a wide linear detection range, a low detection limit and a low value of the apparent Michaelis constant (K_M_^app^), showing an increased affinity of the enzyme for the substrate [80].

### 4.3. Cross-Linking

Enzyme immobilization by cross-linking is an irreversible method achieved by the formation of strong intermolecular bonds between enzyme molecules. The process is accomplished with a cross-linking agent used as a ligand to link enzyme molecules into three-dimensional cross-linked aggregates. Unlike covalent binding, the immobilized enzyme is present in the reaction mixture and does not need to be bound to a specific support. Cross-linked immobilization can be approached in three ways: cross-linked enzyme (CLE), cross-linked enzyme aggregate formation (CLEA) or cross-linked enzyme crystals (CLEC) [144]. In all three methods, a cross-linking agent, such as glutaraldehyde, is used to bind enzyme molecules by reacting free amino groups of lysine residues on the active site of neighboring enzyme molecules.

**Figure 9:**
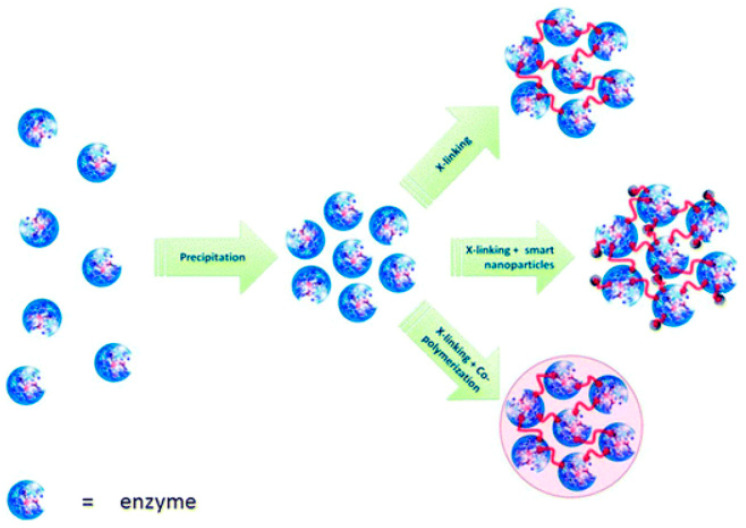
Formation of cross-linked enzyme aggregates (CLEAs) [145].

In the CLEC method, the cross-linking agent is added after crystallization of the enzyme. Immobilized enzymes in CLEC show significant improvements in their mechanical properties, stability and efficiency compared to non-crystallized forms [145,146]. The CLEA method can work in aqueous solutions, whereas CLEC requires the formation of crystals. Another advantage of the CLEA method is the preservation of catalytic activity following the addition of salts, organic solvents or non-ionic polymers for the formation of enzyme aggregates. A schematic of enzyme aggregate formation by cross-linking is shown in Figure 9 [145].

However, there are some disadvantages to these methods, such as poor aggregation due to small molecular sizes. A promising strategy to avoid this limitation would be to capture enzyme aggregates in calcium alginate gels [147,148]. On the other hand, the tendency of molecules to aggregate during centrifugation and filtration processes may hinder their separation and dispersion in solution and thus lower their catalytic activity. Therefore, magnetite cross-linked enzyme aggregates (MCLEAs) represent a way to avoid these limitations [149]. Magnetite nanoparticles show a large contact surface area, and through the silanization reaction, they are activated and can provide a larger binding site for enzyme aggregates. The advantage is that MCLEAs can be easily separated from the reaction mixture using a magnet [150]. The quality of the precipitate depends on the type of enzyme, crosslinker concentration, crosslinking time and mass ratio between the magnetite nanoparticle and the enzyme. Therefore, the optimization of these process variables is mandatory to achieve maximum efficiency (highest enzyme activity, stability, reusability, etc.) [151]. CLEA process optimization can be achieved by Response Surface Methodology (RSM) and experimental design methods, e.g., D-optimal, Box-Behnken [152,153]. İlker Polatoğlu proposed in his study a new CLEA design strategy using stochastic optimization by several direct search methods (differential evolution, simulated recovery, random search and Nelder-Mead) [144].

Since strong covalent bonds of enzyme molecules are formed during cross-linking, enzyme loss is minimal. Another advantage of the method is the possibility to improve the microenvironment for the enzyme by using appropriate stabilizing agents that promote stability [154]. However, the use of glutaraldehyde can lead to changes in the three-dimensional structure of the enzyme and even to loss of activity [21]. For this reason, inert proteins such as gelatin or bovine serum albumin (BSA) can be added during the immobilization process to minimize enzyme damage [135,155,156]. 

Glutaraldehyde is a widely used cross-linking agent for the immobilization of various types of enzymes [157,158] due to its high versatility, excellent reactivity and thermal and chemical stability compared to other aldehydes. The reaction of glutaraldehyde with the primary amino groups in the enzyme and substrate forms a heterofunctional support in which both physical and chemical interactions take place. The binding mechanism of glutaraldehyde allows different approaches to enzyme immobilization [159]. For example, glutaraldehyde has been used for cross-linking tyrosinase and its immobilization on a screen-printed carbon electrode modified with gold nanoparticles. Morphological characterizations confirmed the firm anchoring of the protein groups in the gold nanoparticle lattice, thus forming a strong bond with the enzyme [75]. Additionally, our research group has often used glutaraldehyde cross-linking of tyrosinase on different types of screen-printed electrodes, modified or not with nanomaterials [18,48,160,161], obtaining very good analytical performance and optimal reproducibility. 

Gisela Ibáñez-Redín et al. [82] explored the use of functionalized a black carbon and dihexadecylphosphate surfactant for the construction of an electrochemical biosensor based on tyrosinase immobilized by glutaraldehyde cross-linking for catechol detection. Although they evaluated several cross-linking techniques, the authors chose enzyme cross-linking using glutaraldehyde but also serum bovine albumin. The high intensity of the resulting currents was attributed to the improved electrocatalytic properties following the functionalization of black carbon but also to the catalytic activity of the appropriately cross-linked enzyme through interaction of the cross-linking agent with the carboxyl and hydroxyl groups. Bovine serum albumin was found to protect the activity of the enzyme following cross-linking with glutaraldehyde. The biosensor exhibited a stable response, low detection limit, high sensitivity and high affinity for catechol [82]. 

To improve the electrocatalytic activity of gold electrodes, M. Florescu et al. used cobalt(II)-porphyrin (CoP) as a mediator and enzyme support before the immobilization of tyrosinase by cross-linking with glutaraldehyde. The biosensor was used for dopamine detection by evaluating the dopamine-kinone reduction current obtained by differential pulse voltammetry. The formed matrix favored the efficient immobilization for tyrosinase, thereby decreasing the distance from the redox center of tyrosinase to the CoP film, and promoted faster electron transfer. Similar to the previous study, serum bovine albumin was homogenized in a solution with the enzyme prior to cross-linking. Biosensor performance was evaluated in the presence of dopaminergic medication in biological samples with satisfactory results in terms of recovery (96%) [162].

However, the CLEA strategy offers more opportunities for exploration, for example, for the co-mobilization of multi-enzyme systems, with a cascading reaction taking place [151]. This strategy allows easy diffusion of intermediates between enzymes due to their close proximity to each other [163]. However, the combined CLEA method may not be suitable for certain enzymes, and the stability of the system is always determined by the weaker enzyme [160,161,162]. Therefore, it is important to optimize the operational conditions of the enzymes used in the CLEA system. For example, it is possible to obtain a simple co-aggregation of several enzymes to be captured simultaneously in a single CLEA, and the biocatalytic processes will cascade. Then, the resulting CLEA can be used for a variety of biotransformations.

### 4.4. Entrapment

Immobilization by capture depends on the matrix incorporating the enzyme [162,163,164,165,166,167]. The enzyme can be captured or encapsulated. The difference is that, in the case of encapsulation, the enzyme is encapsulated in a semi-permeable membrane [168]. The membrane acts as a barrier, retaining the enzyme inside but allowing the sublayer and reaction products to pass through. The difference between the capture/encapsulation immobilization technique and the adsorption, covalent and cross-linking methods is that the free movement of the enzyme is restricted by a gel-like or semipermeable membrane network [169].

J. Kochana et al. used a TiO_2_ sol-gel matrix modified with MWCNT, a poly(diallyldimethylammonium polychloride) (PDDA) and Nafion for tyrosinase capture. The amperometric biosensor aimed to detect bisphenol A in a flow-batch monosegmented sequential injection system. Initially, a TiO_2_ sol was prepared by acid hydrolysis and additional polycondensation of titanium isopropoxide. Then, the MWCNT nanocomposite was prepared and dispersed in tyrosinase solution. The mixture of TiO_2_ and polymer was added over the suspension of MWCNT and tyrosinase with vigorous stirring. At the end, the amount of Nafion was gradually incorporated under stirring. Finally, to obtain a homogeneous composite, the resulting mixture was ultrasonicated. For the actual construction of the biosensors, 10 L of nanocomposite was added twice to the surface of the pretreated graphite electrode. The final matrix composition (20 μL/electrode) was exactly calculated, having 0.041 mg MWCNT, 0.039 mg (225 U) TYR, 1 μL of PDDA and 5.7 μL Nafion. SEM characterization of the surface showed that both MWCNT and PDDA contributed to increasing the porosity of the nanomaterial, a feature that favors substrate diffusion through the matrix. The immobilization procedure resulted in very good analytical characteristics of the biosensor, providing accurate results in a short time [170]. 

Another polymer used for tyrosinase incorporation was 1-vinyl-3-ethyl-imidazoliumbromide (ViEtIm^+^ Br^−^). The authors optimized the method to obtain microparticles containing the enzyme captured by the polymerized ionic liquid through a simple anion exchange reaction. Specifically, the microparticles were synthesized after the prior preparation of a concentrated emulsion consisting of 1-vinyl-3-ethyl-imidazolium bromide (poly-(ViEtIm^+^ Br^−^)), and various amounts of bisacrylamide (cross-linking agent) as a discontinuous phase and ammonium persulfate, N,N,N′,N′-tetramethylthylenediamine (polymerization initiators), dodecane and Span 80, as a continuous phase. After a certain reaction time, the polymer precipitated after washing with cold buffer solution. Microparticles were isolated by centrifugation (10 min at 4500 rpm), washed with water and lyophilized. 

Tyrosinase was added to the aqueous solution of the emulsion while keeping the temperature below 20 °C during polymerization in order to prevent denaturation of the enzyme. After this step, the microparticles were lyophilized to remove residual solvent and water. An exact amount of microparticles was added and maintained on the working electrode surface (GCE) using a dialysis membrane. The newly developed amperometric biosensor aimed to detect catechol and other phenolic compounds in domestic water. The analytical technique was cyclic voltammetry and the detection limit (20 μM) and sensitivity (17.95 mAM^−1^ cm^−2^) had low values for catechol detection. Of note, the tyrosinase activity incorporated in poly(ViEtIm^+^ Br^−^) microparticles was unchanged for at least 9 months after their synthesis [171].

Another group of researchers reported a simpler strategy to incorporate tyrosinase using poly(L-DOPA) (a polymer with higher biocompatibility and affinity for tyrosinase than L-DOPA). Immobilization was achieved by in situ capture of tyrosinase in poly(L-DOPA); thus, catalytic polymerization occurred, yielding the composite PD_M_ -Tyr.

The biosensor was prepared by immersing an Au electrode in the PD_M_ -Tyr composite and then coating it with Nafion. It showed excellent performance, superior to most Tyr-based electrochemical biosensors, with a sensitivity of 5122 μA mM^−1^ for phenol detection. 

The low value of the Michaelis-Menten constant (K_M_^app^) indicated strong substrate binding and high catalytic activity of immobilized tyrosinase. The biosensor also performed well with atrazine biodetection in a wide range of linearity (50 ppb and 30 ppm). This biosensor features excellent stability, accuracy, high sensitivity and a simple fabrication technique [172].

The tyrosinase capture method was also used by Chetana Vaghela et al. in their study on the development of an enzyme biosensor for bisphenol detection. Tyrosinase was immobilized by capture in a biopolymer matrix consisting of optimized amounts of agarose, guar gum and graphene oxide. Tyrosinase activity was maintained at approximately 80% after capture and the constructed biosensor recorded an optimal response on bisphenol detection. 

The biosensor was prepared in three steps: (i) formation of a homogeneous graphene oxide suspension and deionized water; (ii) addition of guar gum powder and agarose to the graphene oxide suspension with uniform mixing; and (iii) incorporation of an appropriate amount of extract enzyme. Approximately 50 μL of the resulting solution was added to the surface of the working electrode, represented by a glass plate coated with indium oxide and titan dried at 30 °C. The detection mechanism involved enzymatic oxidation of bisphenols to the corresponding o-bisphenols and, subsequently, their reduction occurring at the active surface of the bioelectrodes. The biosensor proved to be highly selective for bisphenol detection, having stability even after a 150 days of storage [173]. 

Tyrosinase immobilization in polymer films is an advantageous method, as it allows the thickness of the polymer layer and the concentration of the enzyme to be controlled. Furthermore, the incorporation of enzymes into polymer layers can be achieved by electrochemical polymerization of the desired monomer in buffer solutions at a pH higher than the isoelectric point in order to effect electrostatic interactions between the positively charged polaron in the polymer and the negatively charged enzyme. This can be achieved by a number of electrochemical methods, such as potentiostatic, potentiodynamic (cyclic voltammetry) and galvanostatic methods [174,175]. 

C. Lete et al. chose the sinusoidal voltage (SV) method for the electrodeposition of a biocomposite consisting of poly(3,4-ethylenedioxythiophene) and tyrosinase (PEDOT-Ty) on a gold electrode. The biocomposite was deposited in a thin layer on gold-coated quartz crystals. The electrochemical behavior of PEDOT-Ty in aqueous solution was studied by cyclic voltammetry and electrochemical impedance spectroscopy. The authors optimized the parameters used in SV (fixed frequency, high amplitude) for the electrodeposition to take place efficiently, which was confirmed by morphological characterization of the biomaterial by scanning electron microscopy. The prepared biosensor was successfully used for the voltametric detection of dopamine [176].

Over time, it has been observed that the encapsulation of enzyme molecules in Metal-Organic Frameworks (MOFs) improves enzyme stability, favors a high specific surface area, results in high porosity and increases the resistance of biomolecules in less favorable experimental encodings. [177]. However, the low conductivity of most MOFs limits their application [178]. The conductivity of MOFs can be improved by an optimal design of the structure of the organic connectors and the connection to the metal centers. Two-dimensional MOF nanofilms have proven to be excellent materials for building highly efficient electrochemical sensing platforms [179,180,181].

In 2022, Junping Ma et al. [182] tackled the assembly of biomacromolecules into metal-organic frameworks (MOFs) to develop a biosensor for bisphenol A detection. Basically, tyrosinase was deposited in layers in an ultrathin copper-porphyrin MOF nanofilm (Tyr@Cu-TCPP) by a simple solvo-thermal method. 

The synthesis of Cu-TCPP nanofilm MOFs can be explained by connecting the TCPP ligand to four Cu_2_ (COO)_4_ paddle metal nodes, forming stacked layers in the presence of polyvinylpyrrolidone (PVP). The synthesis process involves stabilization of the PVP surfactant and its selective attachment to the surface of the MOFs, resulting in anisotropic growth of Cu-TCPP and the formation of ultrathin stable Cu-TCPP nanosheets [181]. The developed biosensor exhibited excellent performance including a low detection limit and a large linearity range.

This tyrosinase immobilization strategy offers increased thermal and storage stability and tolerance to acidity or basicity compared to the traditional immobilized enzyme [182]. 

### 4.5. Combined Methods of Tyrosinase Immobilization 

Each enzyme immobilization method for enzyme biosensor development has its advantages and limitations. For example, for enzyme immobilization by the covalent binding method, the enzyme can be firmly immobilized, but its conformation can be changed, while by the non-covalent binding method, the enzyme maintains its catalytic activity but can be lost from the support. Therefore, the combination of immobilization techniques can be a very convenient solution to obtain high performance results [183]. This strategy has been applied for the immobilization of tyrosinase on the surface of magnetic amine nanoparticles (Fe_3_O_4_-NH_2_). Specifically, tyrosinase was first adsorbed on the nanoparticle surface by electrostatic interaction. Then, the enzyme fixation was achieved by cross-linking through covalent bonds with glutaraldehyde. The immobilized tyrosinase exhibited enhanced resistance to pH and temperature optimization and significant storage stability. Finally, immobilized tyrosinase was used to screen enzyme inhibitors in 11 traditional Chinese medicines (TCM) [183]. This strategy could be used for the development of enzyme biosensors.

**Figure 10:**
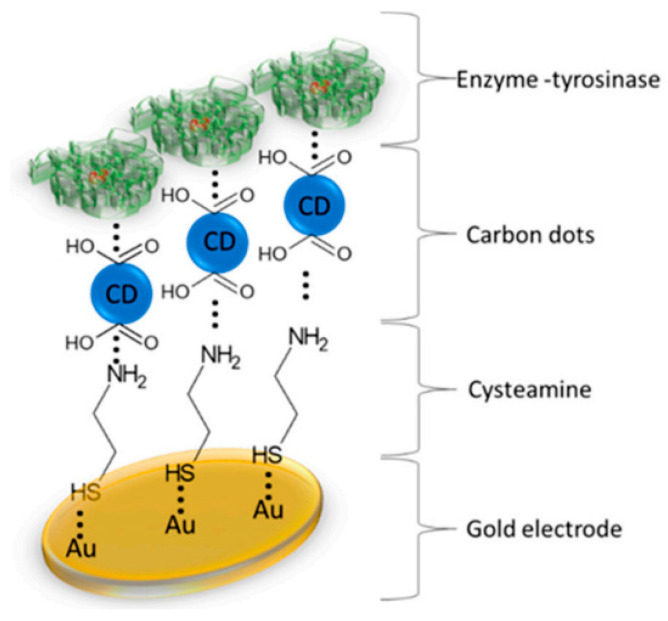
Schematic of the Gold-Electrode/Cysteamine/Carbon Dots/Tyrosinase biosensor [184].

S. Baluta et al. [184] proposed a modified electrochemical biosensor with carbon dots and tyrosinase for the determination of norepinephrine in biological samples. With its simple construction technique, non-toxic nanomaterials and low cost, the biosensor can be portable and useful in the medical industry. Specifically, a gold electrode was modified with a thin layer of cysteamine by physical adsorption, creating thiol bonds. The cysteamine molecule has an amine group, which remains exposed for the next modification step (Figure 10). The carbon dots have negatively charged carboxyl groups which, in addition to stability and good conductivity, also allow interactions to occur with the amine functional groups of cysteamine. Tyrosinase immobilization (at an optimum pH of 7.0) was achieved by physical adsorption, then cross-linked with 10% glutaraldehyde. 

FT-IR spectroscopic analysis confirmed the formation of specific functional groups of carbon dots (required for protein anchoring on the surface of CDs), as well as the formation of the cysteamine-carbon dot-tyrosinase system. The biosensor showed good selectivity and recovery for the voltametric detection of norepinephrine in synthetic urine [184].

Subsequently, the same author proposed a new technique to develop a tyrosinase-based biosensor for the detection of epinephrine in pharmaceutical samples. Specifically, a glassy carbon electrode was electrochemically modified (cyclic voltametric deposition) with a polythiophene derivative (poly-4,40-bis(2-methyl-3-butyn-2-ol)- 2,20-bithiophene, poly-4,40-bBT), acting as a matrix for subsequent tyrosinase immobilization and as an electron transfer mediator. The binding of tyrosinase after electrode modification with the poly-4,40-bBT matrix involved its immobilization by physical adsorption, a simple and inexpensive method, but one which carries a risk of desorption of the biocatalyst during analysis. Similar to previous studies, cross-linking with glutaraldehyde was used to avoid enzymatic losses. The physical adsorption immobilization procedure followed by cross-linking lasted approximately 2 h and 20 min, with no additional activation of the groups required. The polymer film structure and bioplatform formation could be observed by atomic force microscopy.

Two electroanalytical techniques were compared: chronoamperometry and differential pulse voltammetry (DPV). Better LOD and a wider linear range was obtained with DPV; therefore, this technique was used for the sensitivity calculation. The biosensor thus obtained was successfully validated in a pharmaceutical product with a recovery of about 98% [185].

Physical adsorption and tyrosinase cross-linking have been addressed in a recent study to develop an amperometric biosensor also for the detection of epinephrine in pharmaceuticals. The authors report the use of a pyridine-thiophene derivative, 4,7-bis(5-(pyridin-2-yl)thiophen-2-yl)benzo[c][1,2,5]thiadiazole as an immobilizing agent for tyrosinase on the surface of a gold disc electrode. The film obtained by anodic oxidation was shown to work effectively in immobilizing tyrosinase, avoiding denaturation or loss of the enzyme. The tyrosinase-based biosensor was prepared by physical adsorption followed by cross-linking by adding 40 L of 40% glutaraldehyde solution in phosphate buffer. SEM characterization revealed the homogeneous and porous structure of the biosensor, and the obtained results showed good electrocatalytic activity of the enzyme [186].

**Figure 11:**
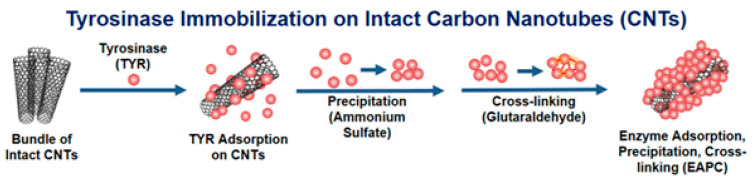
EAPC protocol, consisting of enzyme adsorption, precipitation and crosslinking. Reprinted with permission from [78]. Copyright 2023, copyright Elsevier.

Youngho Wee [78] proposed a novel carbon nanotube and tyrosinase-based biosensor for the detection of phenolic compounds in real water samples by a technique involving adsorption, precipitation and cross-linking of enzyme molecules (EAPC). Ammonium sulphate was used to induce tyrosinase precipitation, and glutaraldehyde was used for cross-linking. By precipitating the tyrosinase molecules, more efficient cross-linking was achieved, resulting in higher enzymatic loading of the CNTs. A schematic of the biosensor development by the EAPC technique can be seen in Figure 11.

EAPC provides 30 times higher enzyme loading than adsorption and 14 times higher when adsorption and cross-linking are used. EAPC modification of a screen-printed electrode (SPE) resulted in better stability and sensitivity than the modification of a glassy carbon electrode [78], probably due to a larger active surface area in the case of SPE.

Finally, Table 2 presents the main characteristics of some biosensors based on tyrosinase, depending on the applied immobilization method and the determined analyte.

As can be seen in Table 2, the detection limit and sensitivity values differ, depending on the immobilization technique but also on the nanomaterials used for functionalization and, of course, on the analyzed analyte. In the case of phenol, the best detection limit was obtained with a biosensor developed by immobilizing tyrosinase by the entrapment method. Catechol and dopamine were detected in a very low concentration with the help of a biosensor developed through the cross-linking technique. So, there is no best immobilization strategy, but only the most suitable strategy for a given transducer, modifying nanomaterial and analyte.

## 5. Conclusions and Future Perspectives

This review highlights the main tyrosinase immobilization techniques for the development of sensitive electrochemical biosensors. Immobilization strategies are classified according to the degree of reversibility/irreversibility of the enzyme binding to the support material and according to the type of method, i.e., physical or chemical. Each method of tyrosinase immobilization has its advantages and limitations, and the selection depends mainly on the type of the support electrode and the nanomaterial modifiers for immobilization. With the capture/encapsulation method, the free movement of the enzyme is restricted by a gel, nanoparticles or a polymer matrix. The retention of the enzyme on the support and the possibility of optimizing the thickness of the polymer layer are important advantages, but the capture/encapsulation strategy involves multiple steps prior to the incorporation of the enzyme such as electropolymerization of the film, improvement of the conductivity of the modifying nanomaterials or the addition of surfactants to increase the stability of the tyrosinase. Therefore, we consider that tyrosinase immobilization by cross-linking presents the best benefit/risk ratio, with the possibility of continuously improving the work steps, the reagents used and the cost of biosensor development.

The CLEA method also favors the dissolution of multi-enzyme systems, leading to portable, multi-functional but also selective and sensitive biosensors. However, we did not find any literature reporting an enzymatic co-immobilization between tyrosinase and another enzyme by the CLEA method for the construction of an electrochemical biosensor. We believe that future research can pursue this approach. The main condition for the co-immobilization and initiation of a cascade reaction is the affinity between the enzymes used and the stability of each of them.

**Figure 12:**
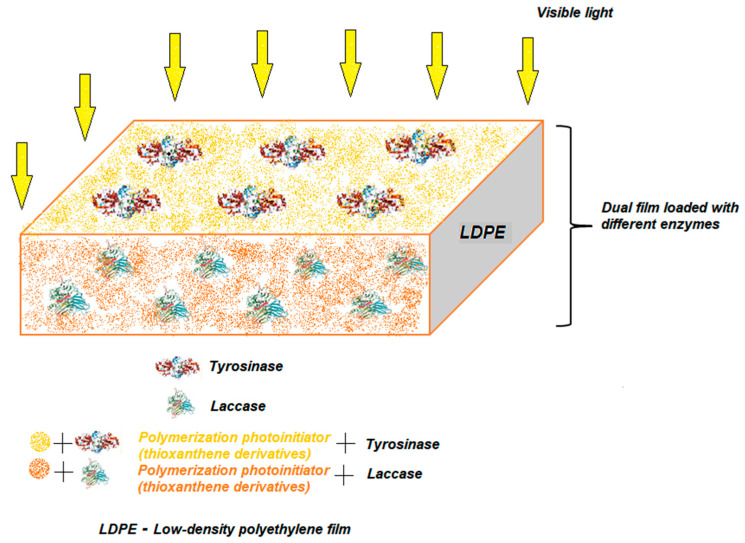
Schematic of the dual enzymatic immobilization process.

Future prospects should also take into account the combination of several immobilization techniques for maximum limitation of enzyme losses, better reproducibility and shorter working time. Besides the formation of aggregates from two or more enzymes, another way of simultaneous immobilization would be the creation of a dual film in which the enzymes are attached by polymerization induced by visible light on one side and the other of the film, without mixing the enzymes. Such a pattern, in the case of two enzymes, is shown in Figure 12. The main advantages of this immobilization strategy would be to avoid, for both enzymes, a loss of the biocatalytic activity and possible non-cooperative interactions between them.

The use of carbon nanomaterials and metal nanoparticles and the optimization of the polymer matrix could improve conductivity and increase the active surface area and porosity of the material.

## Figures and Tables

**Table 1:** Advantages and disadvantages of the main immobilization techniques.

Immobilization Strategy	Advantages	Drawbacks
Physical adsorption	- quick and easy technique - reduced enzyme loss	- desorption or non-specific adsorption - weak or reversible binding between enzyme and carrier
Affinity	- allows the creation of highly ordered structures with high biosensitivity	- the need for the presence of a specific group (e.g., biotin) in a particular enzyme sequence - affinity bonds can affect the biocatalytic activity of the enzyme
Cross-linking	- quick and easy technique - good reproducibility - prevents enzyme loss - no need for a “carrier”	- use of an additional reagent such as glutaraldehyde - cross-linking may decrease the activity of the enzyme due to modification of the three-dimensional structure of the heteroprotein.
Entrapment	- Simple strategy - the activity of the enzyme is not affected by the chemical reaction between the enzyme and the monomer - enzyme can be released through porous matrix, of different types (silica gel, electropolymerized film, carbon paste) - sensitive surface is protected by an anti-contamination effect	- diffusion hindered - risk of enzyme loss - electropolymerization involves high concentrations of enzyme and monomer.
Covalent coupling	- good enzyme diffusion - better stability - quick response time	- high enzyme activity loss - matrix not regenerable - the need for a coupling agent, which can be toxic - multiple stages of preparation

**Table 2:** Detection limit and sensitivity of biosensors based on tyrosinase, depending on the applied immobilization method and the determined analyte.

Biosensor	Immobilization Technique	Analyte	LOD	Sensitivity	Ref.
Biosensor based on tyrosinase onto graphene-decorated gold nanoparticle/chitosan (Gr-Au-Chit/Tyr) nanocomposite-modified screen-printed carbon electrode (SPCE)	Physical adsorption	Phenol	1.6 × 10^−2^ µM	0.624 µA/µM	[113]
Tyrosinase/NiO/ITO biosensor based tyrosinase, nickel oxide nanoparticles deposited on indium tin oxide		Dopamine	1.038 μM	0.0602 µA/µM	[114]
Magnetic loading of tyrosinase-Fe_3_O_4_/mesoporous silica core/shell microspheres		Phenol	1 × 10^−3^ μM	78µA/mM	[115]
Flow-biosensor based tyrosinase and acridine orange-adsorbed carbon felt (TYR/AO-CF)		*p*-Chlorophenol	2.13 × 10^−2^ μM	1.41 µA/µM	[116]
Biosensor based tyrosinase immobilized in a matrix of nanodiamonds and potato starch (Tyr-ND-PS/GCE)	Covalent bond	Catechol	0.39 μM	-	[130]
Tyrosinase modified Au nanoparticles on a screen-printed electrode (Tyr–AuNP–SPE) biosensor		Phenol	0.4994 μM	166.830 μA/µM	[136]
Electrochemical enzymatic biosensor based on polymeric matrix derived from 4-mercaptophenylacetic acid (Poly (MPAA)/CGL)		Tyramine	3.16 μM	-	[137]
Tyrosinase Modified Poly(thionine) Electrodeposited Glassy Carbon Electrode (TYR/GA/pTN/GCE)		Catechol	6.0 µM	5.04 × 10^−3^ µA/μM	[143]
Graphene oxide-modified tyrosinase electrode (TYR/GA/GO/GCE)		Catechol	3 × 10^−2^ μM	-	[80]
Gold electrodes modified with cobalt (II)-porphyrin and tyrosinase(CoP) (CoP-Tyr Biosensor)	Cross-linking	Dopamine	0.43 µM	1.22 µA·cm^−2^/µM	[162]
Screen-printed electrode modified with single-layer carbon nanotubes and tyrosinase (SPE-SWCNT-Ty)		Hydroxytyrosol	3.49 × 10^−2^ μM	-	[48]
Screen printed electrode modified with single-layer carbon-nanotube and tyrosinase (SPE/SWCNT/Tyr)		Oleuropein	9.53 × 10^−2^ μM	0.0718 µA·cm^−2^/µM	[160]
Tyrosinase immobilized on a single-walled carbon nanotube-modified glassy carbon electrode		Epinephrine	2.54 µM	-	[161]
Sol–gel TiO_2_ matrix modified with multi-walled carbon nanotubes (MWCNTs), polycationic polymer poly(diallyldimethylammonium chloride), (PDDA) and Nafion (TYR/TiO2/MWCNTs/PDDA/Nafion)	Entrapment	Bisphenol A	6.6 μM	3.263 µA·cm^−2^/µM	[170]
Electrochemical biosensor based on tyrosinase and ionic liquid polymeric microparticles		Catechol	20 µM	1.795 × 10^−4^ µA·cm^−2^/µM	[171]
Bioelectrode of agarose-guar gum-graphene oxide immobilized with tyrosinase		Bisphenol A	5 µM	2.904 µA·cm^−2^/µM	[173]
Gold electrode based poly(3,4-ethylenedioxythiophene)-tyrosinase (PEDOT-Ty) biocomposite material		Dopamine Hydroquinone	3.9 µM 5.6 µM	-	[176]
Au electrode modified with poly(L-DOPA)-tyrosinase (PDM-Tyr) composite		Phenol *p*-Chlorophenol	2 × 10^−3^ µM 3 × 10^−2^ µM	5.122 μA/µM 1.293 μA/µM	[172]

## Data Availability

Not applicable.
